# Cutaneous cryptococcal infection: Initial manifestation of acquired T-cell immunodeficiency due to malignant thymoma

**DOI:** 10.18502/CMM.8.2.10334

**Published:** 2022-06

**Authors:** Timothy McCann, Anar S. Patel, Neha Patel D, Deepali B. Sharath, Borna Mansouri, Cynthia Contreras

**Affiliations:** 1 Department of Internal Medicine, Good Samaritan Hospital, Cincinnati, Ohio, USA; 2 Department of Infectious Diseases, Good Samaritan Hospital, Cincinnati, Ohio, USA

**Keywords:** Cutaneous cryptococcosis, *Cryptococcus neoformans*, Thymic carcinoma, Type B3 thymoma

## Abstract

**Background and Purpose:**

Cryptococcosis is a known opportunistic infection. Thymomas are known to cause immune dysregulation. We describe an atypical case of cutaneous cryptococcosis in a patient with acquired T cell immunodeficiency that has been found to be secondary to a type B3 thymoma with progression to carcinoma.

**Case report:**

A 63-year-old male presented with a chronic skin lesion confirmed as *Cryptococcus neoformans* on biopsy and an incidental mediastinal mass found during infectious work-up for the notable cluster of differentiation 4 (CD4)+ lymphopenia. This led to the diagnosis of a type B3 thymoma requiring resection. The cryptococcal lesion was treated successfully with azole therapy.

**Conclusion:**

*C. neoformans* is an opportunistic infection rarely associated with isolated T cell immunodeficiency due to thymomas. A multidisciplinary approach and understanding of the pathogenicity of *cryptococcus* and the immunological effect of thymic dysfunction are paramount to diagnosis and treatment.

## Introduction

*Cryptococcus* is a genus of basidiomycetes that comprises over 38 species of encapsulated yeast; however, currently, only *Cryptococcus gattii* and *Cryptococcus neoformans* are considered pathogenic in humans [ 1 ]. *C. neoformans* causes mainly opportunistic infections with widely varied presentations.

Based on the National Cancer Institute’s Surveillance, Epidemiology, and End Results (SEER) Program, the overall incidence of thymoma is 0.13 per 100,000 person-years in the U.S. [ 2 ]. WHO thymoma classification is based on the morphology of the malignant thymic epithelial cells and comprises several histopathological subtypes including type B3 thymomas, which account for 55% of cases [ 3 ]. Type B3 thymomas are well-differentiated carcinomas [ 4 ] composed of epithelioid neoplastic epithelial cells with only a sparse or minimal number of lymphoid cells present that are overshadowed by confluent sheets of epithelioid cells [ 4 ].

While there are case reports of associated opportunistic infections related to thymoma in the literature, a small number have documented cryptococcosis in association with myasthenia gravis or thymoma [ 4 ]. To our knowledge, this is the first case report of localized cutaneous cryptococcosis that has led to the discovery of an isolated CD4+ T cell deficiency secondary to a malignant thymoma type B3.

## Case report

**Figure 1:**
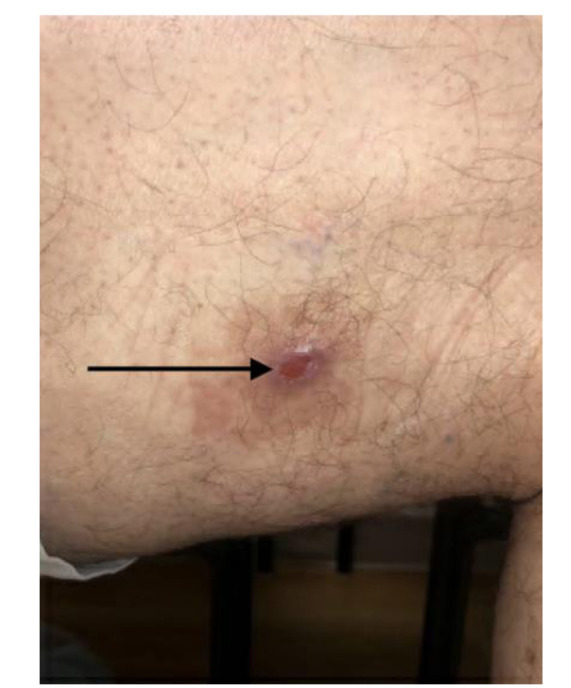
Arrow showing central erosion with surrounding erythema

**Figure 2:**
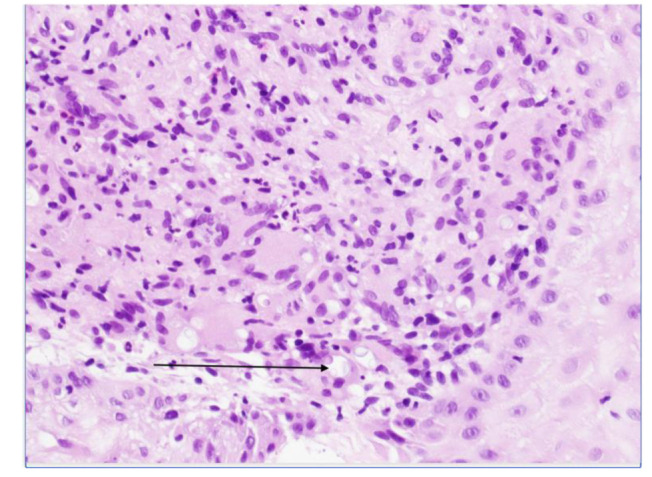
Skin biopsy of the lateral aspect of right thigh skin lesion

**Figure 3:**
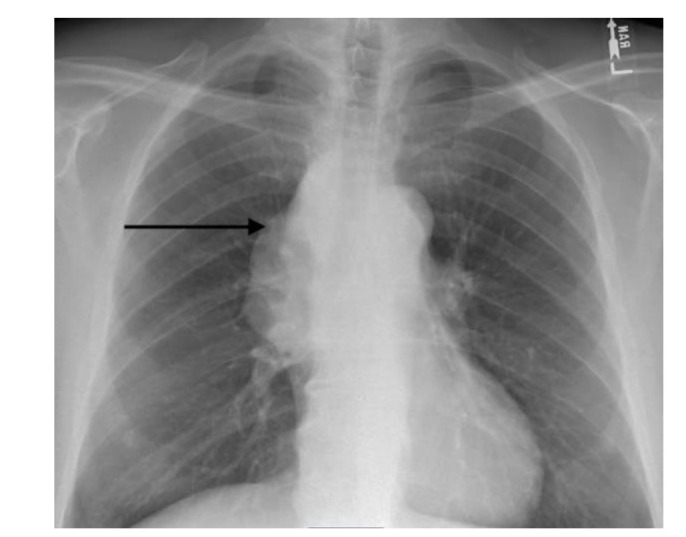
Chest radiograph suspicious for right mediastinal mass

**Figure 4:**
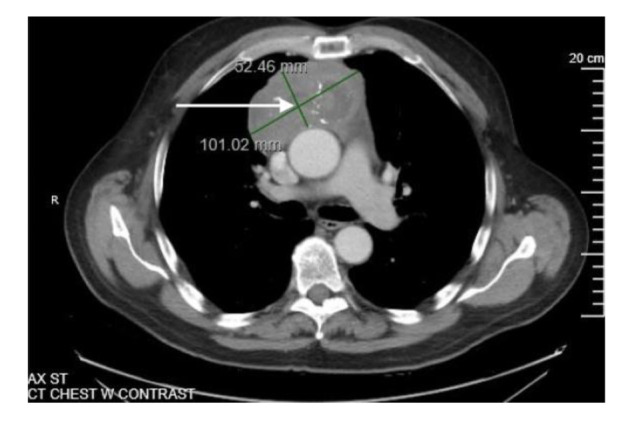
Computed tomography for the thoracic region findings of conglomerate soft tissue and a heterogeneously dense mass with calcifications and cystic components in the anterior mediastinum measuring 10.1 x 5.2 cm extending into the substernal space measuring 10.7 cm superior to inferior

A 63-year-old male with no significant past medical history presented for evaluation of a skin lesion on the lateral aspect of the right thigh existing for six months (Figure 1). He denied any known trauma to the skin and believed that the lesion started as a pimple which he subsequently popped and noted drainage. Over the next few months, he noticed ongoing intermittent drainage and lack of healing along with intermittent pruritus. He was then referred to a dermatologist and underwent a skin biopsy with findings suggestive of cryptococcal infection (Figure 2). The initial work-up indicated causes for possible immunodeficiency (Table 1). A chest radiograph was also performed followed up by computed tomography (CT) with significant findings (Figures 3- 4). Further fungal culture from the skin lesion grew *cryptococcus neoformans* on Remel^TM^ Agar, and no susceptibility testing was performed.^a^ Given image findings and acetylcholine receptor antibodies, the patient underwent a CT-guided needle biopsy of the mediastinal mass (Table 1). 

**Table 1:** Pertinent laboratory results

Test (reference range)	Results
Absolute CD4+ (430-1800 cells/uL)	36 cells/uL
%CD4+ (32-64%)	9%
HIV 1/2 antibody (reactive/non-reactive)	Non-reactive
*Cryptococcal* antigen, serum* (detected/not detected)	Not detected
IgG subclass 1 (240-1118 mg/dL)	643 mg/dL
IgG subclass 2 (124-549 mg/dL)	354 mg/dL
IgG subclass 3 (21-134 mg/dL)	85 mg/dL
IgG subclass 4 (1-123 mg/dL)	118 mg/dL
Acetylcholine receptor antibody (0.0-0.4 nmol/L)	1.2 nmol/L (positive >0.5 nmol/L)

* Cryptococcal Antigen (CrAg) Lateral Flow Assay (LFA) provided by IMMYLabs, reference: CR2003

The final pathology showed a tumor consisting of a mixed epithelial and lymphoid neoplasm with a dense hyalinized fibrous capsule with morphologic features supporting a thymoma type B3 over a thymic carcinoma. After a full informed consent discussion, the patient elected to undergo the oncology recommended chemotherapy with cisplatin, doxorubicin, and cyclophosphamide for four cycles followed by attempted resection. Only partial resection was possible with further pathology showing thymic carcinoma and background thymoma B3. He was then lost to follow-up due to the coronavirus pandemic for six months, but returned, eventually completing radiation with adjuvant chemotherapy with carboplatin and paclitaxel. 

The patient tolerated treatment well. He is currently being monitored off cancer therapy with periodic surveillance imaging. The cutaneous cryptococcosis was treated with oral fluconazole 400 mg daily with a resolution of the lesion after four weeks. He continued suppressive therapy for the duration of chemotherapy. The patient has had no recurrence of cutaneous cryptococcosis.

## Discussion

Localized cutaneous cryptococcosis is a condition in which lesions are confined within a limited part of the skin, not systemically disseminated at the same time, and are associated with neither cryptococcal fungemia nor antigenemia [ 1 ]. Disseminated cryptococcal infection can be present in 10-20% of patients with the cutaneous disease [ 6 ]. This type of secondary cryptococcal disease is most associated with infectious acquired immunodeficiency syndrome (AIDS) but can occur in those with other immunocompromised states including hereditary diseases, cancer, or patients on immunosuppressive medications [ 7 ]. HIV/AIDS in patients with the disseminated cryptococcal disease must be ruled out due to the large incidence associated with the emergence of HIV and the reported increase in cryptococcal disease [ 8 ]. Clinicians must also recognize the life-threatening potential of untreated cryptococcal meningitis and cryptococcal pneumonia in this subset of patients. Even with accurate identification of HIV, treatment of HIV with *cryptococcus* coinfection can lead to deadly cryptococcal immune reconstitution inflammatory syndrome (IRIS) [ 9 ]. 

The presentation of the cutaneous disease is highly variable and includes ulceration, acneiform papules, subcutaneous nodules, and cellulitic disease. Moreover, it can be morphologically indistinguishable from bacterial infection [ 6 , 10 ]. A definitive diagnosis is possible with evidence of encapsulated yeast observed on tissue biopsy. Treatment includes azole derivatives, typically fluconazole 400 mg daily and duration is variable based on the resolution of infection and underlying disease [ 11 ]. 

Immune dysregulation is necessary for cryptococcal infection to occur. CD4+ T cells when severely depleted or absent, allow for cryptococcal infection to flourish. The main adaptive immune mechanism for stimulating B cell maturation and antibody production along with activating macrophages involves multiple CD 4+ T cell subtypes and their respective distinct cytokine production including IFNγ and IL-17 [ 12 , 13 , 14 ]. When absent, there is no production of cytokines, which are critical to an adequate immune response to *cryptococcus* [ 14 ]. 

Immune dysregulation is common with thymomas and thymic carcinomas. An example of a well-established immune dysregulation is myasthenia gravis, which was observed in our patient’s antibody positivity for acetylcholine receptors. Clinically overt immunodeficiency is less common [ 5 ]. Good’s syndrome, the most common associated immunodeficiency syndrome and the first to be described in 1954, encompasses a combination of humoral and cellular immunodeficiency characterized by T cell abnormalities and hypogammaglobulinemia [ 15 ]. Immunodeficient patients with thymomas without hypogammaglobulinemia, as seen in our patient, have “received little attention to date” [ 5 ]. However, reviewers have extrapolated from a few published series of thymoma patients with data on opportunistic infections and T cell function and concluded that an isolated T cell immunodeficiency with thymoma is clinically relevant and probably more frequent than classical Good’s syndrome [ 5 ].

## Conclusion

To our knowledge, no cases have been reported to date of a cutaneous cryptococcosis presentation leading to the diagnosis and treatment of malignant thymoma. Cutaneous *cryptococcus* infections warrant thorough evaluation for possible underlying immunodeficiencies. In thymomas, immune dysregulation is prevalent, isolated T cell immunodeficiency is likely under-reported, and cutaneous cryptococcosis, presenting as a symptom of underlying immunodeficiency from thymoma, has not been reported in the literature. A multidisciplinary approach with specialists in dermatology, infectious diseases, medical oncology, thoracic surgery, radiation oncology pathology, and internal medicine is necessary for the diagnosis and treatment, as highlighted in our case. This is key for identifying both the clinical infection and the underlying pathogenic disease that allows the infection to progress. A well-funded understanding of the immune system and its regulatory effects can help guide accurate diagnosis and timely treatment.

## Acknowledgments

We would like to acknowledge Dr. Megan Smith MD for her contribution to pathologic imaging and Dr. Andrew Parchman MD for his contribution to the pre-print critical appraisal. This report is self-funded.

## Authors’ contribution

T. M., N. P., D. S., B. M., C. C., A. P. contributed to the conception and design of the work. Also, they made significant contributions to drafting and revising the work critically for important intellectual content. They contributed to the final approval of the version to be published and agreed to be accountable for all aspects of the work in ensuring that questions related to the accuracy or integrity of any part of the work are appropriately investigated and resolved. T.M. contributed to the acquisition, analysis, and interpretation of data. 

## Conflicts of interest

The authors declare no conflicts of interest.

## Financial disclosure

This research received no specific grant from any funding agency in the public, commercial, or not-for-profit sectors.

## Ethical Considerations

The authors certify that this manuscript is their original work and has not been previously published elsewhere. All authors mentioned have significantly contributed to the research. All data in the manuscript is authentic and any conflicts of interest have been notified to the Editors. All sources used in the creation of this manuscript have been identified. Written informed consent was obtained. TriHealth Institutional Review Board approval is not needed for case reports.
